# Association between serum uric acid to high-density lipoprotein cholesterol ratio (UHR) and abdominal aortic calcification: a cross-sectional study based on NHANES 2013–2014

**DOI:** 10.3389/fcvm.2025.1560022

**Published:** 2025-05-29

**Authors:** Bowen Li, Yue Xu, Qingwei He, Qiaoxiang Yin, Mengdi Wu, Huijing Zhu, Yanjie Cao

**Affiliations:** ^1^ Graduate School, Hebei North University, Zhangjiakou, Hebei, China; ^2^ The Fifth School of Clinical Medicine, Air Force Clinical Medical School, Anhui Medical University, Hefei, Anhui, China; ^3^ Cardiology Department, Air Force Medical Center, PLA, Beijing, China; ^4^ Graduate School, China Medical University, Shenyang, Liaoning, China

**Keywords:** abdominal aortic calcification, uric acid, high-density lipoprotein cholesterol, serum uric acid to high-density lipoprotein cholesterol ratio, UHR, NHANES

## Abstract

**Background:**

Cardiovascular diseases (CVDs) are among the leading causes of mortality worldwide, with abdominal aortic calcification (AAC) being an independent risk factor. The serum uric acid (sUA) to high-density lipoprotein cholesterol (HDL-C) ratio (UHR) integrates both pro-atherogenic (sUA-induced endothelial dysfunction) and anti-atherogenic factors (HDL-C-mediated cholesterol efflux), which may be associated with vascular calcification. However, epidemiological evidence on their relationship remains scarce.

**Methods:**

This cross-sectional study analyzed data from 2,789 U.S. adults aged ≥40 years in the National Health and Nutrition Examination Survey (*N*HANES) 2013–2014 with complete AAC and UHR data. Participants with invalid AAC imaging, missing sUA/HDL-C measurements, or incomplete calcium/phosphorus intake records were excluded. AAC severity was quantified using the Kauppila scoring system via dual-energy x-ray absorptiometry. UHR was calculated as [sUA [mg/dl] divided by HDL-C [mg/dl]] multiplied by 100. Weighted multivariable linear and logistic regression models assessed associations, while weighted restricted cubic splines explored nonlinear relationships. Subgroup analyses and sensitivity analyses assessed the robustness of findings.

**Results:**

The study included a total of 2,789 participants aged 40 or older. After multifactorial adjustment, the regression model indicated that a higher UHR was significantly associated with increased AAC scores (*β* = 1.055, 95%CI: 1.024–1.087), AAC (OR = 2.605, 95%CI:1.760–3.855), and severe AAC (OR = 2.227, 95%CI:1.649–3.008). The restricted cubic spline analysis revealed significant nonlinear relationships between UHR and both AAC scores and AAC, presenting an inverted “L” shape, with the risk rising sharply at UHR levels close to 17.8–18.0 and then plateauing. Subgroup analyses suggested potential interactions between gender and diabetes in the UHR-AAC association, while sensitivity analyses confirmed the stability of the findings.

**Conclusion:**

In a U.S. middle-aged and elderly population, the UHR was found to be nonlinearly associated with the risk of AAC, and may interact with gender and diabetes. However, due to the cross-sectional design, no causal inferences can be drawn. Future longitudinal studies may be considered to validate these associations and explore whether interventions targeting UHR could potentially slow down the progression of vascular calcification.

## Introduction

Cardiovascular diseases (CVDs) are among the leading causes of mortality worldwide, with an estimated 18.6 million deaths in 2019, accounting for 32% of all global deaths (1). Dysregulation of mineral metabolism, such as calcium and phosphorus, and the deposition of mineralized plaques in the arterial wall, a process known as vascular calcification (2), can lead to the onset and progression of CVDs (3). Abdominal aortic calcification (AAC) is an independent risk factor for cardiovascular events and is closely associated with calcification in other vascular beds and subclinical atherosclerosis (4–6). The Kauppila AAC score, calculated from lateral lumbar dual-energy x-ray absorptiometry scans, ranges from 0 to 24 and is used to assess the severity of AAC, with scores greater than 6 indicating severe abdominal aortic calcification (SAAC) (7, 8).

Serum uric acid (sUA), the end product of purine metabolism, is associated with a variety of diseases. Elevated levels of sUA may increase insulin resistance through mechanisms such as reduced nitric oxide production, endothelial dysfunction, and promotion of vascular smooth muscle proliferation, thereby contributing to the development of atherosclerosis (9, 10). Additionally, increased sUA may also lead to elevated levels of low-density lipoprotein cholesterol (11).

High-density lipoprotein cholesterol (HDL-C) is considered a protective factor against CVDs. HDL-C can modulate low-density lipoprotein cholesterol, thereby reducing the risk of cardiovascular events. Studies have also found that lower levels of HDL-C may be associated with an increased risk of AAC (12, 13).

Notably, sUA and HDL-C exhibit a dynamic antagonistic interaction during arterial calcification. On one hand, sUA promotes premature calcification by activating the NLRP3 inflammasome to induce vascular smooth muscle cell proliferation (14, 15), while on the other hand, HDL-C counteracts this process through reverse cholesterol transport (16). However, current studies predominantly focus on the isolated associations of sUA or HDL-C with AAC (9, 17). In summary, the serum uric acid to high-density lipoprotein cholesterol ratio (UHR) may better reflect the dynamic balance between oxidative stress and anti-inflammatory protection (18–21). To date, no studies have explored the relationship between this ratio and AAC. This study aims to validate the linear relationship between UHR and AAC/SAAC in middle-aged and older adults using data from the National Health and Nutrition Examination Survey (*N*HANES) 2013–2014. These findings may enhance the comprehensive understanding of CVDs and provide novel insights for the prevention, management, and treatment of AAC and SAAC.

## Methods

### Study population

**Figure 1:**
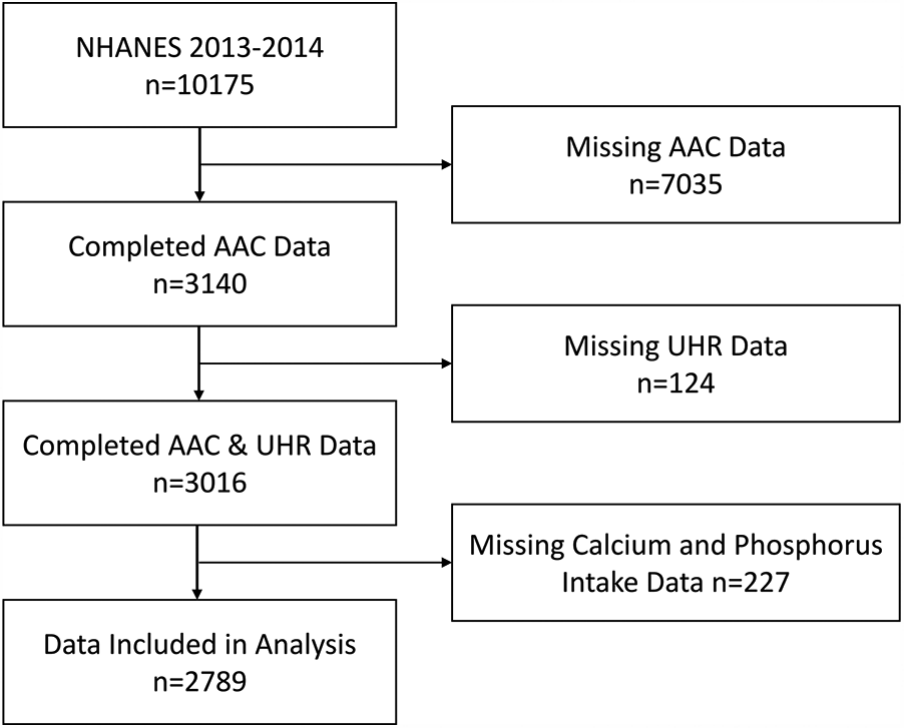
Participant selection flowchart. UHR, serum uric acid-to-HDL cholesterol ratio; AAC, abdominal aortic calcification.

NHANES is a nationally representative survey conducted by the Centers for Disease Control and Prevention in the United States. It employs a stratified, multistage probability sampling method to select participants from across the country, and all data are publicly accessible on the NHANES website (https://www.cdc.gov/nchs/NHANES/index.htm). The National Center for Health Statistics Institutional Review Board approved all NHANES protocols, and all participants provided written informed consent. This study utilized data from a single survey cycle conducted between 2013 and 2014, during which 10,175 participants were surveyed. Since all participants undergoing AAC scoring were aged ≥40 years, we excluded those with invalid data for AAC (*N* = 7,035; including participants aged <40 years, individuals ineligible for DXA scans due to pregnancy, or those with poor image quality; detailed criteria available at https://wwwn.cdc.gov/Nchs/Data/Nhanes/Public/2013/DataFiles/DXXAAC_H.htm), missing UHR data (*N* = 124), and participants missing calcium and phosphorus intake data (*N* = 227). Ultimately, 2,789 participants were included in this study. The specific selection process is depicted in Figure 1.

### AAC and SAAC assessment

AAC scores were quantified using the Kauppila scoring system based on dual-energy x-ray absorptiometry, with examinations performed by trained and certified radiographers at the NHANES Mobile Examination Centers (7, 22). The Kauppila scoring system divides the abdominal aortic wall into four consecutive segments, directly corresponding to the L1 to L4 vertebral regions. Each segment is scored based on the degree of calcium deposition (0–6 points), and the sum of the segment scores constitutes the final AAC score (0–24 points). A total score greater than 6 indicates significant calcification and is classified as SAAC (7, 8). Detailed quantification protocols, including Kauppila scoring quality assurance procedures, are available at https://wwwn.cdc.gov/Nchs/Data/Nhanes/Public/2013/DataFiles/DXXAAC_H.htm.

### UHR measurement

sUA and HDL-C were measured from fasting blood samples collected in the morning. The HDL-C measurement procedure is as follows: Magnesium sulfate/dextran solution is added to the samples to form a water-soluble complex with non-HDL cholesterol, which does not react with the measurement reagents in subsequent steps. Then, by adding polyethylene glycol esterase, HDL cholesterol ester is converted into HDL-C. In the reaction, hydrogen peroxide generated is combined with 4-aminophenazone and N, N-Diethyl-p-phenylenediamine sulfate (HSDA) to form a purple or blue dye. Finally, laboratory personnel determine the HDL level by photometry at 600 nm.

The measurement of sUA is as follows: The DXC800 automated chemistry analyzer uses the timed endpoint method to measure sUA concentration. sUA is oxidized by uricase to produce urocanic acid and hydrogen peroxide. In the reaction catalyzed by peroxidase, hydrogen peroxide reacts with 4-aminophenazone (4-AAP) and 3,5-dichloro-2-hydroxybenzenesulfonate (DCHBS) to produce a colored product, which is then measured at 520 nm to determine the sUA level (23). Subsequently, UHR is calculated by dividing sUA (mg/dl) by HDL-C (mg/dl) and multiplying by 100.

### Covariates definition

Categorical variables included: gender, race/ethnicity, hypertension, diabetes, smoking status, and drinking status.

Continuous variables included: age, family poverty income ratio (PIR), body mass index (BMI), waist circumference, dietary phosphorus, calcium intake, glycated hemoglobin, white blood cell count, alanine aminotransferase (ALT), aspartate aminotransferase (AST), blood urea nitrogen, creatinine, serum phosphorus, serum calcium, total cholesterol, triglycerides, and total bilirubin.

In the home interviews, trained interviewers used computer-assisted personal interviewing to collect demographic information. The following variables relied on self-reporting by participants: age, gender, family PIR, race/ethnicity, smoking status, drinking status, hypertension, diabetes, and dietary intake.

Data obtained from the mobile examination center and laboratory tests included BMI, waist circumference, glycated hemoglobin, white blood cell count, ALT, AST, blood urea nitrogen, creatinine, serum phosphorus, serum calcium, total cholesterol, triglycerides, and total bilirubin.

Smoking status was defined based on the question “Have you smoked at least 100 cigarettes in your life?” and categorized as yes or no. Drinking status was defined through hierarchical criteria: 1) “non-drinkers”: participants answering “No” to Question 1 (“Have you ever consumed at least 12 alcoholic drinks in any one year?”); 2) Current drinkers: those answering “Yes” to Question 2 (“Had at least 12 alcoholic drinks in the past year?”) were classified based on Question 3 (“frequency of alcohol consumption over the past 12 months”) and Question 4 (“number of drinking days per week, month, or year”), calculated into monthly intake and categorized as “1 to <5 drinks/month”, “5 to <10 drinks/month”, and “10+ drinks/month”. Hypertension was defined based on the question “Has a doctor or other health professional ever told you that you have hypertension?” and categorized as yes or no. Diabetes was determined based on laboratory tests indicating “glycated hemoglobin ≥6.5%” or “fasting blood glucose ≥126 mg/dl,” as well as questionnaires asking “Has a doctor ever told you that you have diabetes?” and “Are you currently using insulin?” and categorized as yes or no.

Calcium and phosphorus intake were determined based on both dietary and supplemental sources. Data for both were collected over a two-day period, yet not all participants completed data collection for both days. Since diet is a daily necessity while supplements are not, for participants with dietary calcium and phosphorus intake data for both days, the average intake over the two days was taken; for those with only one day of data, only that day's data was used. For supplemental calcium and phosphorus intake, the average over the two days was calculated. The final calcium and phosphorus intake was the sum of daily dietary intake and daily supplemental intake, defined as mg/day.

### Statistical analysis

Data were analyzed according to the NHANES data analysis guidelines and recommended survey weights. Since there were few missing variables in the overall population, mean imputation was used for missing continuous covariates, and mode imputation was used for missing categorical variables. The missing patterns for each covariate can be seen in Supplementary Table S1. Baseline characteristics were described using unweighted means and weighted standard deviations for continuous variables, and unweighted frequencies and weighted percentages for categorical variables. Baseline characteristics were compared across UHR quartiles (Q1–Q4) using weighted one-way analysis of variance for continuous variables and weighted chi-square tests for categorical variables, with the lowest quartile serving as the reference group (Q1).

Weighted multivariable linear regression models were used to explore the correlation between UHR and AAC scores. Weighted multivariable logistic regression was used to investigate the association between UHR and AAC and SAAC. Additionally, weighted restricted cubic splines (3 knots) were employed to explore potential nonlinear relationships between UHR and AAC. Variance inflation factors (VIF) were calculated to evaluate multicollinearity, confirming the independence of variables (VIF < 10) as detailed in Supplementary Table S2.

To further investigate the relationship between UHR and AAC and SAAC in different population subgroups, subgroup analyses were conducted by age, gender, hypertension, diabetes, and smoking status. The significance of interactions was estimated using the *P* values of interaction coefficients between UHR and subgroup populations. Furthermore, we excluded participants with a history of CVDs (including heart attack, congestive heart failure, angina, and coronary heart disease) from the survey to assess the reliability of our results.

All statistical analyses were performed using R version 4.3.3 (R Foundation for Statistical Computing, Vienna, Austria) and DecisionLinnc version 1.0 (http://www.statsape.com). DecisionLinnc is an integrated software environment that supports multiple programming languages and offers a visual interface for data processing, data analysis, and machine learning (24). Statistical tests were two-tailed, and a *P*-value of less than 0.05 was assumed to indicate statistical significance.

## Results

### Baseline characteristics

A total of 2,789 participants were included in this study. The participants were categorized into four groups based on the quartiles of UHR: Q1 (1.346–7.454), Q2 (7.454–10.213), Q3 (10.213–13.478), and Q4 (13.478–39.583). As shown in Table 1, there were statistically significant differences across UHR groups in terms of AAC, SAAC, gender, tobacco use, alcohol use, diabetes, hypertension, family PIR, BMI (kg/m^2^), waist circumference (cm), calcium intake (mg), glycohemoglobin (%), white blood cell count (10^9^/L), alanine aminotransferase (U/L), blood urea nitrogen (mmol/L), aspartate aminotransferase (U/L), serum creatinine (umol/L), serum phosphorus (mmol/L), serum total calcium (mmol/L), total cholesterol (mmol/L), triglycerides (mmol/L), and total bilirubin (umol/L) (*P* < 0.05). No statistically significant differences were observed across the groups in terms of race/ethnicity, age, and phosphorus intake (mg) (*P* > 0.05).

**Table 1:** Baseline characteristics by UHR quartiles (weighted).

Variables	Overall	Q1	Q2	Q3	Q4	*p*
	*n* = 2,789	*n* = 699	*n* = 696	*n* = 697	*n* = 697	
AAC *n* (%)						0.033
No	1,956 (71.3)	515 (76.5)	501 (71.9)	490 (70.6)	450 (65.7)	
Yes	833 (28.7)	184 (23.5)	195 (28.1)	207 (29.4)	247 (34.3)	
SAAC *n* (%)						0.033
No	2,536 (92.2)	644 (94.1)	645 (92.8)	631 (92.1)	616 (89.8)	
Yes	253 (7.8)	55 (5.9)	51 (7.2)	66 (7.9)	81 (10.2)	
Gender *n* (%)						<0.001
Male	1,341 (48.1)	126 (14.6)	283 (41.5)	393 (60.5)	539 (78.8)	
Female	1,448 (51.9)	573 (85.4)	413 (58.5)	304 (39.5)	158 (21.2)	
Race and ethnicity *n* (%)						0.452
Mexican American	368 (6.9)	78 (5.2)	101 (8.2)	99 (7.5)	90 (7.0)	
Other Hispanic	265 (4.7)	62 (3.9)	78 (5.7)	62 (4.2)	63 (5.0)	
Non-Hispanic White	1,276 (70.9)	343 (74.5)	292 (67.8)	323 (71.5)	318 (69.4)	
Non-Hispanic Black	529 (9.9)	126 (9.2)	130 (10.3)	139 (9.7)	134 (10.4)	
Non-Hispanic Asian	292 (5.2)	73 (5.4)	84 (5.9)	63 (4.6)	72 (5.0)	
Other Race	59 (2.4)	17 (1.8)	11 (2.1)	11 (2.6)	20 (3.2)	
Tobacco use *n* (%)						0.003
No	1,512 (54.5)	422 (59.4)	400 (60.2)	352 (51.9)	338 (46.3)	
Yes	1,277 (45.5)	277 (40.6)	296 (39.8)	345 (48.1)	359 (53.7)	
Alcohol use *n* (%)						0.009
non-drinkers	750 (20.9)	191 (19.3)	213 (25.3)	180 (20.0)	166 (19.2)	
1 to <5 drinks/month	1,441 (52.0)	319 (45.5)	356 (50.7)	367 (54.5)	399 (57.8)	
5 to <10 drinks/month	171 (8.3)	56 (11.7)	26 (5.4)	40 (7.7)	49 (8.0)	
10+ drinks/month	427 (18.9)	133 (23.6)	101 (18.6)	110 (17.8)	83 (15.0)	
Diabetes *n* (%)						<0.001
No	2,195 (83.1)	620 (92.7)	562 (85.3)	525 (79.3)	488 (74.4)	
Yes	594 (16.9)	79 (7.3)	134 (14.7)	172 (20.7)	209 (25.6)	
Hypertension *n* (%)						<0.001
No	1,462 (55.1)	435 (67.0)	387 (57.4)	331 (49.5)	309 (45.6)	
Yes	1,327 (44.9)	264 (33.0)	309 (42.6)	366 (50.5)	388 (54.4)	
Age (years)	58.65 (11.43)	58.09 (11.33)	58.30 (11.33)	58.94 (11.61)	59.29 (11.47)	0.769
Family PIR	2.71 (1.59)	2.89 (1.58)	2.65 (1.62)	2.63 (1.56)	2.66 (1.58)	0.038
BMI (kg/m^2^)	28.55 (5.47)	25.88 (4.94)	28.41 (5.32)	29.35 (4.95)	30.57 (5.13)	<0.001
Waist circumference (cm)	99.54 (13.53)	90.79 (11.85)	98.52 (11.88)	102.20 (11.27)	106.66 (12.03)	<0.001
Calcium intake (mg)	1,055.37 (607.08)	1,145.04 (608.71)	1,049.26 (637.10)	1,037.03 (613.67)	989.91 (547.06)	<0.001
Phosphorus intake (mg)	1,315.12 (548.83)	1,282.96 (515.28)	1,295.10 (545.15)	1,337.80 (571.30)	1,344.69 (557.45)	0.134
Glycohemoglobin (%)	5.91 (0.99)	5.68 (0.83)	5.84 (0.83)	6.01 (1.14)	6.11 (1.05)	<0.001
White blood cell (10^9 ^/L)	7.13 (2.16)	6.57 (1.94)	6.96 (2.10)	7.38 (2.37)	7.62 (2.06)	<0.001
ALT (U/L)	24.70 (18.64)	21.31 (11.34)	24.76 (29.64)	24.69 (13.28)	28.06 (15.12)	<0.001
AST (U/L)	25.55 (15.00)	24.35 (9.02)	25.72 (20.39)	25.12 (10.55)	27.02 (17.80)	0.037
Blood urea nitrogen (mmol/L)	5.12 (2.01)	4.63 (1.56)	4.88 (1.92)	5.19 (2.05)	5.77 (2.40)	<0.001
Serum creatinine (umol/L)	83.27 (34.24)	70.57 (18.22)	79.85 (44.10)	83.73 (26.88)	98.97 (39.02)	<0.001
Serum phosphorus (mmol/L)	1.23 (0.18)	1.27 (0.17)	1.22 (0.18)	1.22 (0.18)	1.20 (0.19)	<0.001
Serum calcium (mmol/L)	2.36 (0.09)	2.37 (0.09)	2.36 (0.08)	2.37 (0.10)	2.36 (0.09)	0.013
Total cholesterol (mmol/L)	5.07 (1.10)	5.25 (0.96)	5.06 (1.12)	5.04 (1.18)	4.93 (1.13)	<0.001
Triglyceride (mmol/L)	1.81 (1.67)	1.17 (0.73)	1.59 (2.43)	1.88 (1.09)	2.60 (1.62)	<0.001
Total bilirubin (umol/L)	10.99 (5.19)	10.68 (4.54)	11.02 (7.24)	11.28 (4.39)	10.98 (4.17)	0.040

UHR, serum uric acid to high-density lipoprotein cholesterol ratio; AAC, abdominal aortic calcification; SAAC, severe abdominal aortic calcification; BMI, body mass index; ALT, alanine aminotransferase; AST, aspartate aminotransferase. Missing continuous covariates were imputed with mean values; missing categorical variables were imputed with mode values.

### The relationship between UHR and AAC and SAAC

As shown in Table 2, the results of the weighted multivariable regression analysis indicated a statistically significant association between UHR and AAC scores, AAC and SAAC, after adjusting for all confounding factors. With AAC score as the outcome variable, the regression coefficient β and its 95% confidence interval (CI) for UHR score were 1.055 (1.024, 1.087). When AAC was the outcome variable, compared to the Q1 group, the odds ratios (OR) and 95%CI for Q2, Q3, and Q4 were 1.691 (1.139, 2.510), 1.772 (1.261, 2.490), and 2.605 (1.760, 3.855), respectively. When SAAC was the outcome variable, compared to the Q1 group, the ORs and 95%CI for Q2, Q3, and Q4 were 1.445 (0.920, 2.270), 1.714 (1.236, 2.377), and 2.227 (1.649, 3.008), respectively. After Bonferroni correction for multiple testing, higher UHR quartiles (Q3 and Q4) showed persistently significant associations with increased risks of both AAC and SAAC, detailed in Supplementary Table S3. The main findings suggest that a higher UHR score may be associated with an increased risk of AAC and SAAC.

**Table 2:** Multivariable regression of UHR and AAC/SAAC.

Outcomes	Model 1		Model 2		Model 3	
	β/OR (95% CI)	*P*	β/OR (95% CI)	*P*	β/OR (95% CI)	*P*
AAC score						
UHR score	1.033 (1.010,1.056)	0.013	1.076 (1.048,1.105)	<0.001	1.055 (1.024,1.087)	0.002
AAC						
Q1	Ref		Ref		Ref	
Q2	1.276 (0.868,1.875）	0.234	1.687 (1.132,2.513)	0.021	1.691 (1.139,2.510)	0.020
Q3	1.356 (0.925,1.988)	0.140	1.970 (1.306,2.972)	0.005	1.772 (1.261,2.490)	0.004
Q4	1.699 (1.305,2.212)	0.001	2.954 (2.104,4.147)	<0.001	2.605 (1.760,3.855)	<0.001
SAAC						
Q1	Ref		Ref		Ref	
Q2	1.248 (0.840,1.855)	0.289	1.588 (1.054,2.394)	0.043	1.445 (0.920,2.270)	0.130
Q3	1.376 (1.016,1.864)	0.056	1.945 (1.299,2.911)	0.005	1.714 (1.236,2.377)	0.005
Q4	1.826 (1.344,2.481)	0.001	2.913 (1.978,4.290)	<0.001	2.227 (1.649,3.008)	<0.001

UHR, serum uric acid to high-density lipoprotein cholesterol ratio; AAC, abdominal aortic calcification; SAAC, severe abdominal aortic calcification; β, regression coefficient; OR, odds ratio; CI, confidence interval.

### Restricted cubic spline analysis

**Figure 2:**
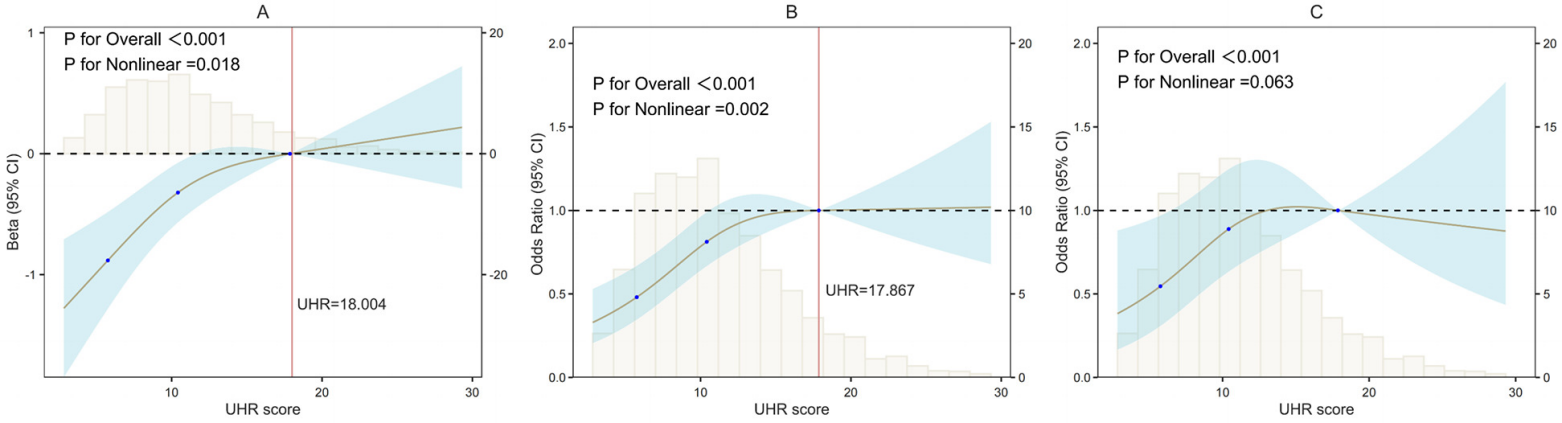
Nonlinear associations of UHR with AAC/SAAC (RCS). **(A)** UHR and AAC scores; **(B)** UHR and AAC; **(C)** UHR and SAAC. UHR, serum uric acid-to-HDL cholesterol ratio; AAC, abdominal aortic calcification; SAAC, severe AAC; β, regression coefficient; OR, odds ratio. *X*-axis, UHR score; *Y*-axis, Statistical estimates (β coefficients or ORs); Black dashed lines: Reference lines (*β* = 0 or OR = 1); Vertical histograms: Data distribution density; Brown solid lines: Restricted cubic spline fits; Blue shading: 95% confidence intervals; Red vertical lines: Inflection points.

The restricted cubic spline (RCS) analysis, as depicted in Figure 2A, revealed a significant nonlinear relationship between the UHR score and the AAC score (P for Nonlinearity = 0.018). The change in the β coefficient approached zero after the UHR score reached approximately 18.004, indicating a saturation point. Figure 2B illustrates a similar significant nonlinear relationship between the UHR score and the presence of AAC (P for Nonlinearity = 0.002), with the odds ratio (OR) stabilizing at 17.867. However, the nonlinear relationship between the UHR score and SAAC, as shown in Figure 2C, was not significant (P for Nonlinearity = 0.063).

### Subgroup and sensitivity analyses

**Figure 3:**
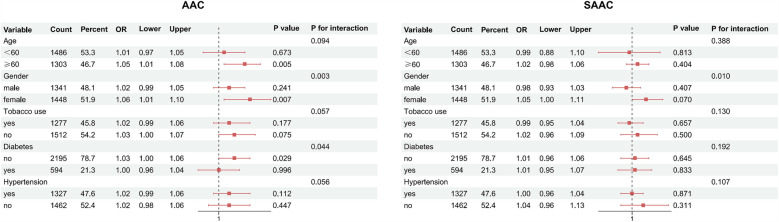
Subgroup analysis of UHR-AAC/SAAC associations (forest plot). AAC, abdominal aortic calcification; SAAC, severe AAC; Red squares, odds ratios (ORs); red solid lines, 95%confidence intervals; black dashed lines, reference (OR = 1).

Subgroup analyses, as shown in Figure 3, indicated that the interaction effects between the UHR score and AAC were statistically significant in terms of gender and diabetes (P for interaction <0.05), while no significant interactions were observed for age, smoking status, and hypertension (P for interaction >0.05). The interaction effects between the UHR score and SAAC were significant for gender; however, no significant interactions were noted for age, smoking status, diabetes, and hypertension. Sensitivity analyses, presented in Table 3, demonstrated that the fully adjusted weighted multivariable regression model results remained robust after excluding participants with a history of CVDs (*n* = 287). Specifically, excluding these individuals did not alter the magnitude of associations between UHR and arterial calcification. For AAC as the outcome, the ORs for Q2–Q4 changed by −3.8%, −2.4%, and +2.4%, respectively, while for SAAC, the OR changes were +1.2%, +3.0%, and −4.8%. Confidence intervals overlapped substantially, and statistical significance (*p* < 0.05) was preserved for Q3 and Q4 in both models.

**Table 3:** Sensitivity analysis of UHR-AAC/SAAC (excluding CVDs history).

Statistical measures	AAC score	AAC				SAAC			
	UHR score	Q1	Q2	Q3	Q4	Q1	Q2	Q3	Q4
β/OR	1.055	Ref	1.627	1.730	2.667	Ref	1.462	1.765	2.120
95%CI	1.024,1.087		1.104,2.398	1.178,2.540	1.654,4.301		0.896,2.384	1.108,2.810	1.333,3.629
P	0.002		0.026	0.013	0.001		0.149	0.030	0.007

UHR, serum uric acid to high-density lipoprotein cholesterol ratio; AAC, abdominal aortic calcification; SAAC, severe abdominal aortic calcification; CVDs, Cardiovascular diseases; β, regression coefficient; OR, odds ratio; CI, confidence interval.

## Discussion

Our study findings indicate a significant association between higher UHR scores and increased AAC scores, as revealed by the RCS analysis, which identified a significant nonlinear relationship characterized by an inverted L-shaped curve and a saturation point at a β coefficient of 18.004. Compared to the lower UHR group, the higher UHR group exhibited a markedly elevated risk of AAC, and similarly, a significantly heightened risk of SAAC. The RCS analysis showed a significant nonlinear relationship between UHR and AAC risk, with a saturation point for the OR value at 17.867. Although the nonlinear association between UHR and SAAC risk was not statistically significant, the inverted L-shaped curve suggests a potential threshold effect, where SAAC risk reaches a plateau at higher UHR levels (e.g., Q4). This pattern is consistent with findings from a Japanese cohort study (20). The borderline significance (*P* = 0.063) may reflect the smaller sample size of the SAAC subgroup (*n* = 253) or residual confounding by unmeasured factors. Future studies with larger samples and comprehensive metabolic profiling are warranted to validate this trend. Subgroup analyses suggest that the results may interact with gender and diabetes, and sensitivity analyses confirm that the UHR association with AAC and SAAC is consistent with the overall findings.

Recent studies in China first proposed the clinical value of UHR in diagnosing abdominal aortic aneurysm (AAA), finding that AAA patients exhibited significantly higher UHR levels than controls. These findings suggest UHR may independently predict AAA in Chinese populations and serve as an auxiliary screening tool in clinical practice (25). Other research demonstrates UHR's significant positive correlation with insulin resistance (IR), indicating superior predictive accuracy for IR compared to isolated sUA or HDL-C measurements, positioning UHR as a potential IR biomarker in U.S. populations (26). Concurrently, Turkish studies identified UHR as a predictor of left main coronary artery stenosis (27), while acute coronary syndrome cohorts revealed UHR's superior predictive value over LDL-C for culprit plaques (19). Collectively, these findings highlight UHR's dual role in atherosclerotic plaque instability and medial calcification. Observed discrepancies may arise from tissue-specific responses to UHR-driven oxidative stress and inflammation. Japanese studies further identified a nonlinear association between UHR and brachial-ankle pulse wave velocity (baPWV), with sex-specific interactions suggesting UHR correlates positively with arterial stiffness in females through a saturation effect, a pattern absent in males (20). This aligns with our subgroup findings, potentially attributable to sex hormone variations in female participants.

Studies demonstrate that elevated estrogen levels are associated with reduced cardiovascular disease risk (28). Postmenopausal women exhibit significantly elevated sUA levels (29), which has been established as an independent predictor of cardiovascular events in this population. HDL-C, a well-documented anti-atherosclerotic factor, shows positive correlations with female sex hormone levels (30, 31). Menopause induces complex hormonal and metabolic shifts, leading to substantially increased cardiovascular disease incidence, with earlier menopause onset correlating with poorer cardiovascular outcomes (32, 33). In diabetic patients, hyperglycemia-induced vascular endothelial damage and insulin resistance likely contribute to elevated AAC risk. Chronic hyperglycemia disrupts endothelial integrity and increases vascular permeability, creating a microenvironment conducive to calcium deposition (34, 35). Progression to diabetic nephropathy further exacerbates vascular calcification through renal dysfunction-mediated phosphorus-calcium metabolism dysregulation (36, 37). Chinese diabetic patients with elevated UHR show independent associations with cardiorenal complications (38), paralleling our findings of heightened AAC risk in diabetics. This suggests possible synergistic interactions between hyperglycemia and UHR pathways in exacerbating vascular calcification.

sUA promotes endothelial dysfunction via oxidative stress and inflammatory cascades, potentially mediating the UHR-AAC association observed in our study. Specifically, elevated sUA reduces nitric oxide bioavailability by enhancing superoxide generation and impairing endothelial nitric oxide synthase activity (39–41). This oxidative stress fosters a pro-calcific milieu within vascular walls. The nonlinear UHR-AAC relationship may indicate threshold mechanisms where sUA-induced NF-*κ*B activation (42) overwhelms physiological defenses, accelerating monocyte-endothelial adhesion and vascular mineralization—critical processes in AAC progression.

HDL's protective role against AAC may become saturated at higher UHR levels, as suggested by our nonlinear, inverted L-shaped association. The functional impairment of HDL likely involves the following pathways: impaired reverse cholesterol transport (17, 43), enabling lipid accumulation in aortic walls; compromised anti-inflammatory capacity (44), permitting cytokine-mediated calcification; and defective antioxidant activity (45, 46), allowing uric acid-driven oxidative vascular damage. This triad of functional deficits explains why AAC risk plateaus despite rising UHR, providing a rationale for clinical risk stratification thresholds.

In summary, UHR demonstrates potential as a cardiovascular risk assessment indicator. However, its precise mechanistic role in AAC pathogenesis remains unclear. We expect that our findings may provide valuable insights for AAC risk management and prevention. Nevertheless, several limitations of this study warrant consideration. First, the cross-sectional design precludes causal inference and only establishes exposure-outcome associations. Second, the exclusion of a substantial number of participants due to invalid AAC measurements may introduce selection bias. Third, self-reported variables are susceptible to recall bias, and residual confounding from unmeasured factors (e.g., inflammatory biomarkers, renal function, physical activity) cannot be excluded. Fourth, the absence of sex hormone measurements (e.g., estradiol, testosterone) and detailed menopausal staging limits our ability to explore gender-specific mechanisms in depth. Finally, the generalizability of our findings to non-U.S. populations requires further validation.

## Conclusion

Based on an analysis of NHANES data, this study uncovers a significant correlation between the UHR and an elevated risk of AAC and SAAC within the middle-aged and elderly population in the United States. The results indicate a nonlinear relationship between UHR and the incidence of AAC, with the risk plateauing at a UHR threshold of around 17.9. Additionally, gender and diabetes status emerge as potential influencing factors that may modify this association. These findings position UHR as a promising biomarker for evaluating the risk of AAC. If this correlation is validated in prospective studies, the identified UHR threshold could prove invaluable in identifying high-risk individuals during routine clinical screenings. Future research should concentrate on longitudinally validating this threshold, exploring strategies to regulate UHR levels, and conducting risk assessments in specific subpopulations (e.g., gender, diabetes) and across ethnicities. Ultimately, these endeavors could contribute to the development of targeted prevention strategies aimed at reducing the cardiovascular disease burden associated with AAC.

## Data Availability

The raw data supporting the conclusions of this article will be made available by the authors, without undue reservation.
